# Transmissible Viral Proventriculitis in Broiler Chickens from Bosnia and Herzegovina

**DOI:** 10.3390/pathogens14050438

**Published:** 2025-04-30

**Authors:** Jovana Dervović, Šejla Goletić, Alma Šeho-Alić, Senad Prašović, Teufik Goletić, Amer Alić

**Affiliations:** Veterinary Faculty, University of Sarajevo, Zmaja od Bosne 90, 71000 Sarajevo, Bosnia and Herzegovina; jovana.supic@vfs.unsa.ba (J.D.); sejla.goletic@vfs.unsa.ba (Š.G.); alma.seho.alic@vfs.unsa.ba (A.Š.-A.); senad.prasovic@vfs.unsa.ba (S.P.); teufik.goletic@vfs.unsa.ba (T.G.)

**Keywords:** transmissible viral proventriculitis, broiler chickens, histopathology, PCR, CPNV, CAV, GyV3

## Abstract

The etiology of transmissible viral proventriculitis (TVP) of broiler chickens has been discussed since its initial recognition 40 years ago. Regardless of its low direct impact on mortality rate, it leads to high economic losses in the broiler industry through reduction of food conversion, weakening of birds, and their increased susceptibility to pathogens. The aim of the present study was to examine the potential presence of TVP on the broiler chicken farms in Bosnia and Herzegovina, to characterize microscopic lesions, and to investigate the viruses implicated in etiology of TVP by PCR-based methods. In total, 143 diseased broiler chickens from 16 farms in Bosnia and Herzegovina were euthanized and subjected to necropsy and subsequent histopathology of proventriculi. A representative number of proventriculi samples (*n* = 50) that exhibited histopathologic changes were processed for molecular detection of chicken proventricular necrosis virus (CPNV), girovirus (GyV3), chicken anemia virus (CAV), and infectious bursal disease virus (IBDV) by PCR-based methods. In addition, samples of bursa of Fabricius (*n* = 39) and spleen (*n* = 50) were tested for IBDV. Histopathology revealed changes consistent with TVP in 39.8% (57/143) and LP (lymphocytic proventriculitis) in 2.1% (3/143) of samples. All 50 proventricular samples showed positivity to CPNV with Ct values ranging between 18 and 26. GyV3 was detected in eight samples (16%), with Ct values ranging from 11.1 to 27.5. The presence of CAV was more prominent (38%), with 19 positive broiler chickens (Ct ranging from 9.6 to 35.6). Pooled samples of spleen, bursa, and proventriculi from three farms were positive for IBDV. The obtained results represent the first documented data on TVP and the first record of CPNV and GyV3 presence in broiler farms from Bosnia and Herzegovina.

## 1. Introduction

Transmissible viral proventriculitis (TVP) is a disease of broiler chickens that has low direct mortality; however, poor food conversion and malabsorption leads to weakening and growth reduction, the main reasons for economic losses, which highlight the need for prompt diagnosis confirmation [1].

Clinical signs of TVP are not specific and include poor feathering, stunted growth, and diarrhea with particles of undigested food [2]. The definitive diagnosis is based on gross lesions (swollen and fragile proventriculi, prominent papillae, and dilated proventricular glands) and histopathology that reveals necrosis of oxynticopeptic cells, ductal hyperplasia, and intraglandular lymphocytic infiltrates [1,2,3,4].

Since its first report in the Netherlands in 1978 [5], when the infectious nature of the disease was suggested, the etiology of TVP has been widely discussed, and some viral agents have been proposed [1,5,6,7,8,9,10,11,12,13]. Among these, only a few have been associated with the disease, with chicken proventricular necrosis virus (CPNV) being the most commonly reported [3,4,14,15,16,17].

First, adenovirus-like particles were observed in the proventricular tissue of the animals with TVP signs, but they stayed unclassified for much longer [2]. In 2005, the virus (named AdLV R11/3) with the same characteristics was isolated, and it has demonstrated a few morphological differences from the *Adenoviridae* family, which prove that this virus is distinct from known adenoviruses [1]. Due to tropism for proventricular tissue and resulting lesions, this newly recognized non-enveloped, double-stranded RNA virus was initially referred to as CPNV and was finally identified as a novel birnavirus, morphologically distinct from other birnaviruses [11]. In addition, gyrovirus (GyV3) and cyclovirus were recently isolated from broilers showing lesions similar to those observed in TVP induced by CPNV [12,13]. Furthermore, successful experimental reproduction of TVP was induced by GyV3 [18] with lesions similar to those caused by CPNV. In addition, GyV3, like chicken anemia virus (CAV), expresses an immunosuppressive effect on chicken immunity. It has been proven that co-infections with these two viruses cause more severe immunosuppression than mono-infections [19]. Moreover, the GyV3 infection in chickens is important from the perspective of public health due to its zoonotic potential [20].

Due to the large negative indirect effects on the broiler chicken industry, TVP should be regularly screened for at farms, and all suspected cases should be thoroughly investigated. Even though broiler farms regularly experience malnutrition, stunted growth, and underperformance (personal communications with farmers), no reports on TVP in Bosnia and Herzegovina have been published. To close this knowledge gap, the aim of this study was to examine the potential presence of TVP on the broiler chicken farms in Bosnia and Herzegovina, to characterize microscopic lesions, and to investigate the viruses implicated in the etiology of TVP by PCR-based molecular methods.

## 2. Materials and Methods

### 2.1. Sample Collection

A total of 143 broiler carcasses (Table 1) from 16 farms (three to 12 broilers per farm) in Bosnia and Herzegovina (BIH) were collected for gross and microscopic examination for the presence of TVP characteristic lesions. Selected flocks were located in three cantons of the Federation of Bosnia and Herzegovina (FBiH), with most flocks originating from Tuzla Canton. The ages of sampled carcasses ranged from 7 to 41, and the majority were 21 to 28 days old (Table 1). Sampled broiler chickens showed stunted growth and were poorly feathered with signs of diarrhea. At necropsy, from each carcass, samples of one half of the proventiculi (*n* = 143), spleens (*n* = 143), and bursa of Fabricius (*n* = 143) and all parenchymatous organs that showed morphological changes were collected for histopathology. The samples were fixed in 10% neutral buffered formalin for 24–48 h and routinely processed for histopathology through a series of alcohols and xylol and finally embedded into the paraffin blocks. Thin sections of four to six micrometers cut on a microtome (Myr STP 120, Tarragona, Spain) were stained with hematoxylin and eosin (H&E) and examined under a light microscope (Olympus BX51, Hamburg, Germany). In parallel, the other half of the proventricular, splenic, and bursal tissue was frozen at −20 °C for further molecular investigation for the presence of CPNV, GyV3, CAV, and infectious bursal disease virus (IBDV).

All the samples of proventricular tissue (*n* = 143) examined under the light microscope were assessed for the five key histopathological features (glandular lymphocytic infiltration, ductal epithelial hyperplasia or metaplasia, necrosis of epithelial cells, luminal glandular dilation with or without sloughing of cells, and cellular infiltrates in lamina propria). The first three were previously designated characteristics for TVP [14]. These parameters were divided in four categories, namely – (absence), + (up to 10 glands), ++ (up to 50% of glands), and +++ (more than 50% of glands) based on the analysis of whole slides at 10X, 20X, and 40X magnifications.

Based on observed histopathological changes, results are divided into the three following groups: broilers with no TVP/lymphocytic proventriculitis (LP) changes, broiler chickens with TVP changes, and broiler chickens with LP changes (only lymphocyte infiltrates presented). Furthermore, all TVP or LP positive samples (*n* = 50) were subjected to PCR-based detection of CPNV, IBDV, and gyroviruses (GyV3 and CAV).

### 2.2. Nucleic Acid Extraction

The frozen halves of proventriculi that showed clear histopathological changes (*n* = 50) were subjected to total nucleic acid extraction and PCR-based detection methods. In addition, total nucleic acids were extracted from frozen spleen samples (*n* = 50) from seven farms and bursa of Fabricius (*n* = 39) from six farms. These samples were treated with proteinase K as described in the DNA Purification from Tissues protocol of the QIAamp DNA Mini Kit (Qiagen, Hilden, Germany); all samples were incubated in a water bath at 56 °C for three hours and were vortexed briefly every 15 min. After proteinase K treatment, they were briefly centrifuged, and 300 µL of each sample supernatant were used for automated nucleic acid extraction with an NX-48S Viral NA Kit on a Genolution Nextractor^®^ NX-48S (Genolution, Seoul, Republic of Korea) according to the manufacturer’s instructions. Any remaining tissue samples were stored at −80 °C, and extractions were stored at −20 °C until further analysis.

### 2.3. CPNV Reverse Transcriptase Quantitative PCR (RT-qPCR)

The proventricular samples (*n* = 50) from seven farms were analyzed with CPNV RT-qPCR. The samples were first pooled in 25 pools, each consisting of two individual samples originating from the same farm (Table S1). If the pools tested positive, both samples in the pool were considered positive.

The primers used for CPNV RT-qPCR are the same as in Guy et al. [11]. Before commencing with RT-qPCR reactions, the 5 µL of pooled samples were first incubated at 95 °C for two minutes and then frozen at −80 °C for 90 s. Viral cDNA was produced and amplified using the SuperScript™ III First-Strand Synthesis System (Thermo Fisher Scientific Inc, Waltham, MA, USA): the first reaction consisted of 5 µL of (thermally treated) pooled samples, 1 µL (0.2 µM) of B2R primer, 1 µL of 10 mM dNTP mix, and 3 µL of DEPC-treated water. The reaction was incubated at 65 °C for 5 min and then at −80 °C for 1 min. The products of the first reaction were added to the second reaction, which consisted of 2 µL 10× RT buffer, 4 µL of 25 mM MgCl_2_, 2 µL of 0.1 M DTT, 1 µL of RNaseOUT (40 U/µL), and 1 µL of SuperScript III RT (200 U/µL). This mixture of reactions was incubated at 50 °C for 50 min, then at 85 °C for 5 min, and then at −80 °C for 1 min. The product of this reaction was used as a sample for downstream qPCR analysis.

The final RT-qPCR reaction consisted of 10 µL of SsoFast™ EvaGreen^®^ Supermix (Bio-Rad, Hercules, CA, USA), 0.6 µL (0.3 µM) of B2F primer, 4.4 µL nuclease-free water, and 5 µL of the sample obtained through previous (upstream) PCR reactions. Thermocycling was performed on a Mic qPCR cycler (Bio Molecular Systems, Sydney, Australia), consisting of initial denaturation for 30 s at 98 °C, followed by 40 cycles of denaturation for 5 s at 95 °C, annealing for 30 s at 56 °C, and extension for 30 s at 65 °C, followed by a melting point analysis consisting of one cycle of incubation for 1 min at 95 °C and subsequently increasing the temperature from 70 °C to 95 °C, with an increment of 0.5 °C/cycle. Fluorescence signals were measured and collected at the end of each extension step.

### 2.4. Gyrovirus-Specific Multiplex Quantitative Real-Time PCR (qPCR)

For gyrovirus-specific qPCR, we used a multiplex approach, which enabled the detection of the chicken anemia virus (CAV) and gyrovirus homsa 1 (GyV3) in the same reaction. A total of 50 proventricular samples were analyzed with gyrovirus-specific multiplex qPCR (Table S1).

The primers used in the multiplex reaction for the detection of CAV and GyV3 qPCR are the same as in Yang et al. [19]. The specific primer and probe mix was prepared, consisting of 4 µM 100 pmol/µL of each primer and probe. The final multiplex qPCR reaction consisted of 6.25 µL of nuclease-free water, 2.5 µL of 5× QuantiFast Pathogen Master Mix (Qiagen, Hilden, Germany), and 1.25 µL of specific primer and probe mix (final concentration 0.4 µM). Thermocycling was performed on a Mic qPCR cycler (Bio Molecular Systems, Sydney, Australia), consisting of initial denaturation for 5 min at 95 °C, followed by 40 cycles of denaturation for 15 s at 95 °C, annealing for 30 s at 62 °C, and extension for 30 s at 50 °C. Fluorescence signals were measured and collected at the end of each extension step on FAM and CY5 channels; the CAV-specific probe was detected on an FAM channel, whereas the GyV3-specific probe was detected on a CY5 channel.

### 2.5. IBDV Reverse Transcriptase Quantitative PCR (RT-qPCR)

For IBDV RT-qPCR testing, samples of spleen, bursa, and proventriculus were separately pooled into 20 pools (seven pools of spleen, six pools of bursa, and seven pools of proventriculi) according to the farm origin (seven farms) (Table S2). In order to separate the chains of IBDV dsRNA, the 5 µL of pooled samples were first incubated at 95 °C for two minutes and then frozen at −80 °C for 90 s. After this, they were added to the 20 µL mastermix consisting of AgPath-ID™ One-Step RT-PCR Reagents (Thermo Fisher Scientific Inc., Waltham, MA, USA), prepared according to the manufacturer’s instructions. The primers and probes used to detect IBDV were the same as in Tomás et al. [21], which enable the discrimination between very virulent and classic/variant IBDV strains. The final concentration of each primer in the mastermix was 0.9 µM, whereas the final concentration of each probe was 0.2 µM. Thermocycling was performed on a Mic qPCR cycler (Bio Molecular Systems, Sydney, Australia), consisting of reverse transcription for 10 min at 48 °C, inactivation of reverse transcription for 10 min at 95 °C, followed by 40 cycles of denaturation for 15 s at 95 °C and annealing/extension for 1 min at 60 °C, and a final step for 5 min at 70 °C. Fluorescence signals were measured and collected at the end of each annealing/extension step.

## 3. Results

**Figure 1:**
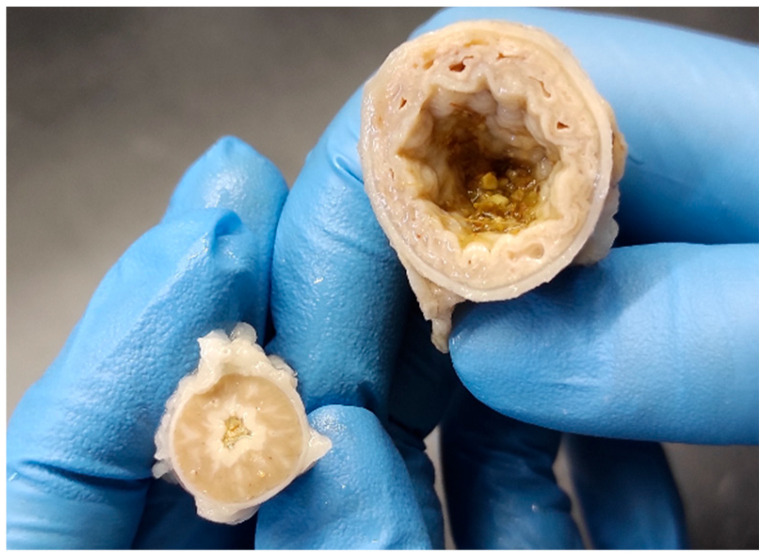
Broiler chicken, proventriculus: a severely enlarged and dilated proventriculus of a TVP affected broiler chicken (**upper right**) in comparison with the proventriculus of a normal unaffected broiler chicken (**lower left**).

On the gross exam, there was marked dilation of the proventriculus. On the cut section, glandular dilation and a few white-to-gray foci were also evident (Figure 1) in five examined broiler chickens.

**Figure 2:**
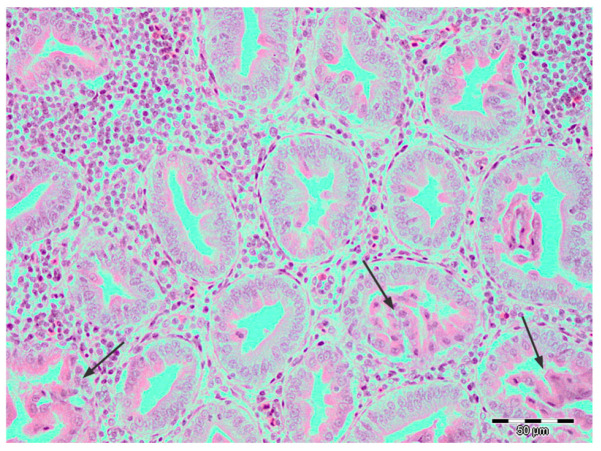
Broiler, proventriculus: diffuse infiltration of lymphocytes and plasmacytes and segmental necrosis (arrows) of hyperplastic alveolar epithelium of proventricular glands of a TVP-affected broiler chicken. Hematoxylin and eosin, scale bar 50 µm.

**Figure 3:**
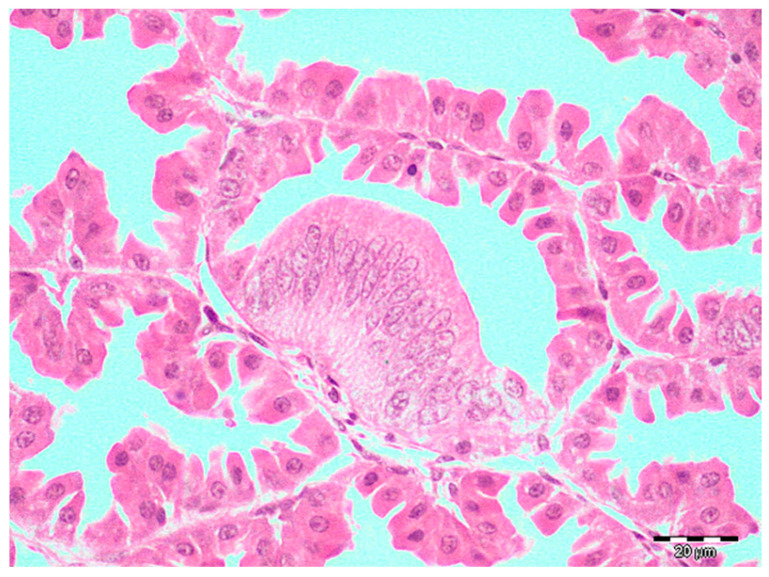
Broiler, proventriculus: a focus of segmental metaplasia of the alveolar epithelium of proventricular glands of a TVP-affected broiler chicken. Hematoxylin and eosin, scale bar 20 µm.

Histopathology revealed changes consistent with TVP in 39.8% (57/143) and LP in 2.1% (3/143) samples. Lymphocytic infiltrates were predominantly distributed focally or rarely multifocally, randomly in the glandular interstitium, whereas diffuse, severe forms were observed in only 12 samples (Figure 2). These cases were accompanied by differing degrees of necrosis. Ductal hyperplasia was present in almost all samples with lymphocytic infiltrates (54/57), sometimes accompanied by degenerative or necrotic changes in the epithelium of the newly formed ductus. Foci of metaplasia were more rarely observed than hyperplasia and were usually present as foci of unilateral change (Figure 3).

Only three samples were characterized as LP due to the sole presence of lymphocytic infiltrates.

Other lesions included mild to moderate gland mineralization, interstitial edema, dilatation of submucosal glands, mild interstitial fibrosis, and mild heterophilic intraglandular or mucosal infiltration.

Dilation of the submucosal glands was present in almost every sample without obvious association with severity of TVP changes. Similarly, as previously stated [22], mostly moderate lymphocytic infiltrates observed in the lamina propria were considered normal findings. Thymic atrophy was noted in two and atrophy of bursa of Fabricius was noted in five broiler chickens.

The results of PCR testing for CPNV, GyV3, and CAV are presented in Table S1. All 25 pools of proventricular samples showed positivity to CPNV, with Ct values ranging between 18 and 26 and average Tm values of 82.2 ± 0.3, and they originated from all sampled flocks but one (7 days old). GyV3 was detected in eight samples (16%) originating from three different flocks, with Ct values ranging from 11.12 to 27.55. The presence of CAV was more prominent, with 19 positive broilers (38%) originating from seven flocks (Ct ranging from 9.62 to 35.64). All three viruses were found together in eight samples, of which only three showed severe TVP changes.

Testing for IBDV was positive in pooled samples of spleen, bursa, and proventriculus from three farms. Out of a total of 20 sample pools, nine were identified as positive for the classic/variant IBDV strain (Ct ranging from 20.64–33.9), and only one was identified as positive for the very virulent IBDV strain (Ct 23.55) (Table S2). The pool that tested positive for the very virulent IBDV strain consisted of spleen and bursa samples from the same farm, and, interestingly, the proventriculi sample pool from the same farm tested negative for any IBDV strain whatsoever. Subsequent RT-qPCR testing of separate samples from nine pools positive for the classic/variant IBDV strain (a total of 47 samples) found all but three proventriculus, bursa, and spleen samples testing positive to the same strain (Ct ranging from 18–36).

## 4. Discussion

Transmissible viral proventriculitis is a disease that causes significant economic losses in modern poultry production. Even though it has been known for over four decades, the main etiological factors implicated in the pathogenesis of the disease still remain unclear [3,4,11,14,17,23]. In Bosnia and Herzegovina, and even in the Balkan region, this ever-growing problem is mainly underreported, and there are no data available on the disease.

Traditionally, TVP is diagnosed primarily based on histopathological lesions observed in proventricular tissue, and all proventriculi with lymphocytic infiltrates, ductal hyperplasia or metaplasia, and presence of necrosis are categorized as positive cases [14,23]. On the other hand, the cases with the presence of lymphocytic infiltrates only are interpreted as lymphocytic proventriculitis. Based on this division, the results of our research suggest that distribution of this disease in our country is similar to that in Poland [17], United Kingdom [3], Brazil [4], and USA [24]. In addition, we identified the disease in all broiler flocks, which signals that it is widespread in the country’s poultry production. Nevertheless, final conclusions and assumptions about the presence and seasonality of the disease should be reached by research stretching through a longer time frame [3].

The most significant lesions in the TVP-positive cases in the current study were lymphocytic infiltrates, mainly in the follicular form, followed by ducal metaplasia and hyperplasia. Necrosis was the least scored lesion. These findings are in accordance with previous papers, with the exception of the severity of necrosis observed [3,4,14,17,24]. Here, the cells featuring necrotic changes such as cytoplasmic hypereosinophilia or karyopyknosis, which were still attached to the basement membrane, were considered necrotic. The cells of similar morphology sloughed in the lumen of glands were considered a normal finding due to the fact that these cells are often observed in normal healthy proventricular tissue as a consequence of physiological replacement of glandular tissue. The epithelium, especially in the digestive system of all animals, is prone to degenerative changes soon after death [25,26,27]; hence, it is expected to see a degree of degenerative changes in the cells of the proventricular glands. Right after euthanasia, we attempted to minimize these effects by fast immersion of the samples into the formalin. However, despite this, we observed aggregates of sloughed cells in the glands of TVP-free samples.

In our study, we commonly observed chromatin marginalization in attached and sloughed cells with or without cytoplasmic hypereosinophilia; however, like in many of the previous studies [4,14,17], intranuclear inclusion bodies were not present. On the other hand, in one study [3], multiple intranuclear eosinophilic bodies, suspected to be viral inclusions, were detected in the oxyntopeptic cells aggregated in the alveolar lumens of 22% of TVP-positive cases. Nevertheless, in only one of these cases was the CPNV infection confirmed. Similar inclusion bodies have also been noted in two other instances [2,28]. The observation of intranuclear inclusions in TVP samples negative for CPNV indicates that other viruses might potentially be implicated in pathogenesis and the development of TVP lesions.

We detected necrotic foci mainly in proventriculi affected with diffuse lymphocytic infiltrates, previously described as an acute stage of the disease [23]. In addition, all three cases of lymphocytic proventriculitis can be considered as being in the chronic stage, as previously suggested [3,4,17].

As expected, we failed to find histopathological changes in one flock, considering the young age of the broiler chickens (7 days), due to the fact that the youngest reported age of natural infection is 18 days. Likewise, the age of one of our flocks exceeded the age frame previously reported (18 to 31 days); however, this group represents resampling of the same flock after 20 days, with the aim of observing differences in the degree of lesions. We detected a slight decrease in the necrotic score, while those for infiltrates and ductal hyperplasia/metaplasia remained the same, which is consistent with aforementioned changes related to chronicity. Unfortunately, possible regression of the disease or eventual re-emergence of the process in the same flock can only be discussed in experimental studies where all the factors, including the broiler chickens’ life span, are controlled [12].

Despite great efforts to elucidate the etiology of TVP, there is no single causative agent that can be identified with certainty as the main or only factor of this disease. Among all viral agents implicated in its etiology, CPNV, GyV3, and cyclovirus are the only ones confirmed in the affected proventricular tissue alone, with CPNV being most commonly reported [1,12,13,14,19].

In our research, we confirmed the presence of CPNV in the TVP-affected proventriculi. All 25 pools of samples were tested positive for CPNV, which is consistent with previous reports [3,4,14,17,23,24] that this virus is responsible for the occurrence of TVP throughout the world. We cannot discuss the prevalence of this virus in our country, but low Ct values and the fact that this virus is present in all RT-qPCR-examined flocks suggest the wide spread of the virus in broiler chicken farms in Bosnia and Herzegovina. Insight into its real distribution can only be acquired through examination of proventriculi from affected and healthy broiler chickens of the same region.

Multifactorial etiology of TVP has long been investigated in many studies, and various viruses have been identified and debated as potential causative factors [5,6,7,8,9,10,12,13,23,29]. In our study, we used qPCR to search for the presence of two more gyroviruses (CAV and GyV3), which have recently been associated with TVP etiology [12,19]. These viruses, as well as other potential viral causes, have not always been included in the design of previous TVP studies [3,4,17,24]. Our results confirmed the presence of both viruses, with CAV being more commonly detected than GyV3. Moreover, to the best of our knowledge, this is the first report of the concurrent presence of CPNV and GyV3 in the tissue of proventriculi with TVP lesions. Apart from CPNV, GyV3 is the only virus with an experimentally confirmed role in the pathogenesis of TVP [12,18]. Infection with GyV3 is persistent and results in aplastic anemia, immunosuppression, and lymphocytic inflammation in various tissues, including proventriculus [18]. The dominance of GyV3 was more recently confirmed among the isolated genomes of the virome of TVP-positive chickens in China [30]. On the other hand, CAV is not associated with the development of TVP lesions but is a widespread pathogen of broiler chickens worldwide and like GyV3 causes severe aplastic anemia and immunosuppression [31]. Synergistic immunosuppressive effects of these two viruses were tested in an experimental model of specific pathogen-free chicks, where severe lymphocytic depletion in thymus, spleen, and bursa of Fabricius followed by hyperplasia of connective tissue were observed [18]. No similar pathology was observed in our study; however, in three cases of severe TVP, which were positive for all three viruses (CPNV, GyV3, and CAV), the potential immunosuppressive effect of GyV3 and CAV could not be excluded. In another five cases of coinfection with all three viruses, observed mild TVP lesions could be attributed to the interaction of other modulatory factors such as immunity and feed quality [4,9,32].

Our results represent the first report of coinfection with GyV3 and CAV in broiler flocks in Bosnia and Herzegovina. The immunosuppression in broiler chicken flocks associated with the spread of CAV across Europe was observed in Bosnia and Herzegovina and neighboring countries in the 1980s and early 1990s; nevertheless, serious studies on these two viruses are very rare [33]. Based on a small number of samples, almost 100% prevalence of CAV was noted by in situ hybridization in archived samples from broiler chicken flocks from Slovenia and Croatia [33]. Two decades later, widespread natural CAV infection in parent flocks along with antibody titers in 26.6% of healthy broiler flocks were documented in Croatia [34]. In the rest of the neighboring countries, data on CAV or GyV3 infection in broiler chickens or parent flocks are missing.

In our study, samples of bursa of Fabricius, proventriculi, and spleen of broiler chickens from three farms were RT-qPCR positive for IBDV; however, no lesions compatible with infectious bursitis were observed. Based on the lack of lesions, it is challenging to ascertain the possible effects of this virus on the immune status of the investigated broiler chickens. Furthermore, from the anamnestic data, it is visible that all the sampled flocks were vaccinated against IBDV, including the so-called “hot strain” of IBDV (E228), which, especially in immunocompromised individuals, may cause latent or clinically manifested infection. It is known that an adequate and timely vaccination program against IBDV can provide a satisfying level of protection even against CAV infection based on the interference between the two viruses [34].

The results of the present study are insufficient to assess the true contribution of GyV3 to the development of TVP of infected broiler chickens. However, the fact that it is present in flocks in Bosnia and Herzegovina and its known zoonotic nature and adaptability to different hosts [20] are very concerning from the point of public health. Poultry is directly labeled as the source of GyV3 infection in outbreaks with severe clinical problems in adults and children [35]. Poultry meat is a significant protein source worldwide [36], and the same holds true for Bosnia and Herzegovina, where the poultry meat, for the most part, comes from domestic production [37]. The detection of GyV3 in 25% of the broiler chicken flocks investigated in this work represents a starting point for further studies of its distribution in the country’s poultry farms. Furthermore, its distribution in populations of other domestic and wild animals, and also in humans, should be the focus of future studies.

We are aware that our study has several limitations. First of all, our financial resources have limited our ability formolecular testing of all broiler chickens for implicated viruses. We tested only the proventricular samples with TVP lesions. Furthermore, due to the pooling of samples, we could not express a true prevalence for CPNV or express the correlation between histopathology and PCR results. In addition, financial constraints prevented us from sequencing PCR products for phylogenetic analysis. Last but not least, the samples with observed lesions could have been subjected to immunohistochemistry or in situ hybridization, at least for CPNV and GyV3, in order to discern the true association of these viruses with the TVP lesions. However, we believe that the results of our study are valuable because they represent the first data on pathology and etiology of TVP in broilers from Bosnia and Herzegovina and also the wider Balkan area. The classical lesions of TVP such as lymphocytic infiltrates and glandular hyperplasia were consistent pathologic findings; nevertheless, our observation of necrosis brings to mind the necessity of more precise characterization of this pathologic entity in proventricular tissue, at least in the cases of TVP. The multifactorial nature of TVP etiology is corroborated, which merits further studies on this complicated problem in poultry production.

## Figures and Tables

**Table 1:** Identification and the location of the farms (*n* = 16) of origin of the broiler chickens (*n* = 143) with the number of sampled animals per farm and their age.

Town/Location of the Farm	*n* of Sampled Broiler Chickens	Age (Days)
Kalesija *	1. Floor—10	21
	2. Floor—5	21
Kalesija	9	15
Gračanica	4	22
Stjepan Polje	10	15
Kalesija *	8	41
Tešanj	8	7
Tarčin	11	21–22
Tarčin	6	34–35
Gradačac	5	31
Gradačac	3	31
Gradačac	5	31
Gradačac	5	31
Gradačac	5	31
Brijesnica Mala, Opština Doboj istok	1. Floor—10	20
	2. Floor—12	16
Piskavica, Opština Gračanica	1. Floor—5	23
	2. Floor—6	23
Tolisa, Opština Orašje	1. Floor—5	21
	2. Floor—5	21
Ilijaš	6	34

* The same farm sampled in two intervals.

## Data Availability

The original contributions presented in this study are included in the article/Supplementary Materials. Further inquiries can be directed to the corresponding author.

## Supplementary Materials

The following supporting information can be downloaded at: https://www.mdpi.com/article/10.3390/pathogens14050438/s1, Table S1: Sampled proventriculi (*n* = 50) of broiler chickens and results of molecular testing for CPNV, CAV and GyV3; Table S2: The results of molecular testing for classical and virulent IBDV in pooled samples of proventriculi (*n* = 50), bursas (*n* = 39) and spleens (*n* = 50) of investigated broiler chickens.
