# Supplementary material for: Transmissible Viral Proventriculitis in Broiler Chickens from Bosnia and Herzegovina
Source: Pathogens. 2025 Apr 30;14(5):438. doi: 10.3390/pathogens14050438 (PMC12113904; doi:10.3390/pathogens14050438)
Supplement: Supplementary file 1 [file pathogens-14-00438-s001.zip › pathogens-3473952-supplementary.pdf]

Table S1: Sampled proventriculi (*n*=50) of broiler chickens and results of molecular testing for CPNV, CAV and GyV3.

Farm ID	Broiler chicken ID	Proventricular sample ID	PCR results (ct vaules)		
			CPNV	CAV	GyV3
139/22	A	15P	18	-	-
	C	17P	22.66	-	-
	D	14P	22.84	34,29	-
	F	16P	18	-	-
101/21	A	48P	24.71	26,32	27,49
	B	43P	24.14	-	-
	C	45P	26.53	9,62	-
	D	49P	25.03	-	-
	E	47P	24.71	20,64	21,3
	F	46P	26.53	15,76	-
	G	50P	25.03	-	-
	H	44P	24.14	-	-
203/21	3	40P	24.95	-	-
204/21	2	41P	26.46	-	-
205/21	2	51P	23.72	32,53	-
206/21	3	52P	23.72	35,24	-
207/21	2	42P	26.46	-	-
88/22	A I 1	5P	26.12	-	-
	A I 3	11P	22.29	-	-
	A I 4	3P	23.19	-	-
	A II 1	13P	22.84	-	-
	A II 3	8P	22.42	-	-
	A II 4	6P	26.12	-	-
	A II 9	1P	25,12	-	-
	C I 3	2P	25,12	-	-
	C I 5	12P	22.29	-	-
	C II 3	9P	24.27		
	C II 5	10P	24.27		
181/21	1	19P	24.72	34,53	-
	2	22P	23.93	34,15	-
	4	20P	24.72	34,21	-
	5	21P	23.93	33,74	-
	6	18P	22.66	-	-
	10	24P	23.32	17,54	-
	11	23P	23.32	14,26	-
182/21	1	26P	25.65	11,32	12,68
	2	28P	25.18	28,15	27,44
	3	29P	23.4	-	-
	4	27P	25.18	27,38	26,87
	5	25P	25.65	10,56	11,95
	6	30P	23.4	-	-
82/21	A5	35P	25.52	35,64	11,12
	A6	33P	25.2	-	-
	A8	39P	24.95	-	-
	A9	32P	23.1	-	-
	A11	34P	25.2	-	-
	A13	31P	23.1	-	-
	A14	36P	25.52	32,47	27,55

	B6	38P	25.66	-	-
	C 2-1	37P	25.66	-	-

Table S2. The results of molecular testing for classical and virulent IBDV in pooled samples of proventriculi (*n*=50), bursas (*n*=39) and spleens (*n*=50) of investigated broiler chickens.

No	Tested tissue	Pool No	Samples in the pool	Result (ct)	
				Classical IBDV	Virulent IBDV
1	Proventricul	1	1P – 13P	-	-
2		2	14P – 17P	32.2	-
3		3	18P – 24P	-	-
4		4	25P – 30P	32.01	-
5		5	31P – 39P	-	-
6		6	40P – 42P, 51P, 52P	34.88	-
7		7	43P – 50P	33.9	-
8	Bursa	8	1B – 5B	33.62	-
9		9	6B – 12B	-	-
10		10	13B – 18B	20.64	-
11		11	19B – 27B	-	-
12		12	28B – 35B	23.23	-
13		13	36B – 39B	22.83	-
14	Spleen	14	1S – 12S	-	23.55
15		15	13S -17S	-	-
16		16	18S – 26S	-	-
17		17	27S – 30S	29.16	-
18		18	31S – 36S	26.97	-
19		19	37S – 43S	-	-
20		20	44S – 50S	30.51	-
